# Investigation of the physiological and molecular regulatory mechanism of soluble sugar metabolism in *Lavandula angustifolia* Mill. under cold stress

**DOI:** 10.3389/fpls.2025.1537516

**Published:** 2025-07-08

**Authors:** Yuchen Liang, Yinan Liu, Yu Wang, Ruijiao Yang, Zening Yuan, Hui Li

**Affiliations:** ^1^ Key Laboratory of Aquatic Biodiversity Research in HeiLongjiang Province, Harbin Normal University, Harbin, China; ^2^ Heilongjiang Provincial Key Laboratory of Plant Biology in Ordinary Colleges and Universities, Harbin Normal University, Harbin, China; ^3^ Institute of Botany, Chinese Academy of Sciences, Beijing, China; ^4^ Botanical Garden, Institute of Botany, Chinese Academy of Sciences, Beijing, China

**Keywords:** *Lavandula angustifolia* Mill., cold stress, sugar metabolism, key genes, molecular docking

## Abstract

Lavender (*Lavandula angustifolia* Mill.) is a valuable aromatic plant with significant commercial importance. However, cold stress–one of the primary abiotic factors impacting sugar metabolism–adversely affects its agricultural productivity in Northeast China. To investigate the mechanisms underlying cold tolerance in *L. angustifolia* and support economic development, we measured the sugar content and performed transcriptome analysis at temperatures of 30°C (control), 20°C, 10°C, and 0°C. The results revealed that when the temperature dropped from 30°C to 0°C, the amylase activities and the content of maltose and glucose increased, while the starch content decreased. During the process, the up-regulation of *LaAMY* and *LaBAM1/3* suggests an adaptive response in *L. angustifolia* to cold stress by promoting the breakdown of starch. Meanwhile, the up-regulation of sugar metabolism genes *LaRHM1*, *LaMUR4*, *LaUGD4*, alongside the downregulation of photosynthesis-related genes *LaPSAD1*, *LaPSAN*, *LaPSBQ2*, *LaLHCB4.2*, and *LaPSB27-1* illustrate a strong connection to soluble sugar metabolism. These key genes exhibit significant correlations with starch content and amylase activities, specifically in the decomposition of starch into soluble sugars. The results indicate the decomposition of starch into soluble sugars plays a crucial role in osmotic regulation, facilitating subsequent sugar metabolism in *L. angustifolia* under cold stress. The correlation between gene expression and physiological indicators suggests that genes can potentially mitigate light-induced damage while promoting cellular homeostasis. Molecular docking analyses between the proteins PSAN and RHM1, MUR4 and UGD4, as well as between LHCB4.2 and RHM1, MUR4, and UGD4 predict that these protein interactions involved in sugar metabolism and photosynthesis contribute to enhancing cold resistance in *L. angustifolia*.

## Graphical Abstract

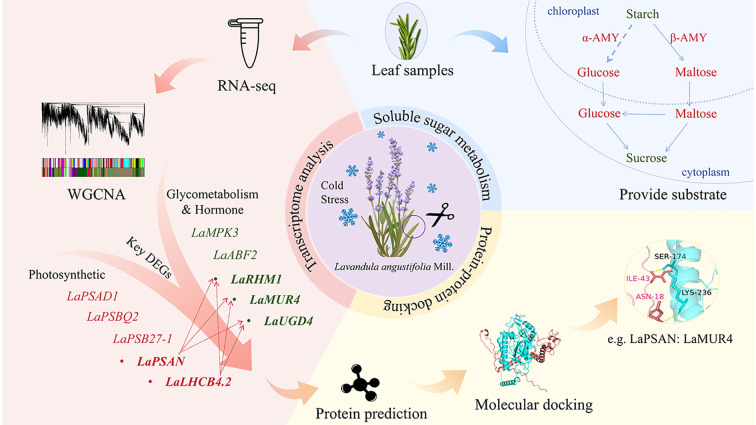

## 1 Introduction

Cold stress is caused by sudden temperature drops in autumn and unexpected cold snaps in late spring. For most plants, cold stress can be categorized into two types: chilling stress (below 20°C) and freezing stress (below 0°C) (Dou et al., 2016). The damage induced by cold stress significantly affects growth, health, and distribution limits of plants and causes more worldwide economic losses to agriculture than any other climate-related hazard (Lamichhane, 2021). Plants have undergone intricate evolutionary processes, equipping them adaptive strategies to survive in cold environments (Meng et al., 2025). It is well-documented that starch metabolism can facilitate adaptive changes in source-sink carbon allocation, thereby helping plants withstand cold stress (Dong and Beckles, 2019). Thus, investigating the regulatory mechanisms of starch metabolism underlying plants responses to cold stress has significant implications for agricultural productivity and biodiversity conservation.

The predominant soluble sugars, including glucose, maltose, and sucrose, are crucial for transporting and storing organic materials in both “source” and “sink” tissues of plants. Importantly, accumulation of sugars such as sucrose and glucose under cold stress is thought to protect cellular structures from freezing damage and regulate osmotic pressure. For instance, sucrose acts as a cryoprotectant by reducing the damage of cell membranes at low temperatures and scavenging reactive oxygen species (ROS) (Bai et al., 2023). Meanwhile, the starch and sucrose metabolism involves various enzymes, including β-amylase (BAM), α-amylase (AMY), sucrose synthase (SUS), and starch synthase (SS). Modifying the activities of these enzymes can accelerate the starch-sucrose pathway and enhance soluble sugars formation at low temperatures (Zhang et al., 2017a). Additionally, a study has revealed a significant increase in the levels of glucose and rhamnose in plants when subjected to cold stress (Ghabel and Karamian, 2020). Thus, an investigation of changes into the soluble sugar metabolism under cold stress conditions is beneficial for enhancing plants cold tolerance.

Sugar signals are multifunctional molecules in plants that connect photosynthetic metabolism with cold stress regulation. The regulation of photosynthesis is intricately linked to the metabolism of sugars in plants (Rozpądek et al., 2019). Photo inhibition and light-induced damage can adversely affect energy synthesis and metabolism in plants, especially under cold stress (Guo et al., 2024). This provides a substantial explanation for the effects of low temperatures on sugar metabolism (Hassan et al., 2021). Thus, the stability of the photosynthetic system (PS) at low temperatures is of significant importance. During the process of photosynthesis, energy can be harvested by the light-harvesting chlorophyll a/b binding protein (LHCB) in photosystem II (PSII) (Zhang et al., 2022). The proteins photosystem II subunit Q2 (PSBQ2) and LHCB4.2 in PSII are also crucial for capturing light energy and maintaining the stability of PSII (Wu et al., 2021; Ojosnegros et al., 2022). The photoremediation function of photosystem II subunit Psb27 (PSB27) in PSII is associated with a plant’s ability to adapt to low temperatures. Meanwhile, proteins such as photosystem I subunit D (PSAD) and photosystem I subunit N (PSAN) in photosystem I (PSI) also enhance the efficiency of photosynthesis, with PSAN helping plants acclimate to low temperatures by reducing the formation of ROS (Blanc et al., 2012; Renard et al., 2020).

The starch-sucrose metabolism makes key functions of providing vital substrates for soluble sugar metabolism (Yu et al., 2024). Key genes exert crucial regulatory roles in these metabolic pathway. The synthesis of UDP-rhamnose (UDP-Rha) is catalyzed by the rhamnose synthase (RHM), utilizing UDP-glucose (UDP-Glc), as observed in the substrate of peaches and petunias (Gu et al., 2020; Zhao et al., 2020; Sang et al., 2022). UDP-glucose dehydrogenase (UGD) has been found to convert UDP-Glc to UDP-glucuronic acid (UDP-GlcA) within the UDP-glucose pathway, leading to increased sugar accumulation levels in bamboo and sugarcane (Yang et al., 2020; Mason et al., 2023). UDP-GlcA also serves as a precursor for the synthesis of arabinose (Ara), which can be synthesized from UDP-xylose (UDP-Xyl) through the regulation of the UDP-D-xylose 4-epimerase1 (*MUR4*) (Dugard et al., 2016; Saez-Aguayo et al., 2017). Additionally, under cold stress conditions, *LHCBs* positively regulate guard cell signaling in response to abscisic acid (ABA) by modulating ROS homeostasis in *Arabidopsis* (Xu et al., 2012). Low temperatures increase ABA levels, activating the downstream ABA response promoter ABA-responsive element binding factor 2 *ABF2* gene and initiating the ABA-dependent pathway (Chen et al., 2020). Moreover, sugar signals integrate multiple hormone signaling pathways to regulate the overall growth of plants (Chen et al., 2021). The MAP kinase kinase (MKK3) cascade is also involved in mediating the negative feedback regulation of ABA signaling (Mao et al., 2024). Low temperatures trigger the transport of Ca^2+^ and activates the MAPK cascade, facilitating soluble sugars accumulation and enhancing plant resilience under cold stress conditions (Zhang et al., 2017b).

*Lavandula angustifolia*, a shrub belonging to the Lamiaceae family, is native to the Mediterranean region (Dobros et al., 2023). The flowers and leaves of *L. angustifolia* are rich in polysaccharides and other antioxidants usually used in the production of high-value cosmetics, essential oils, and medicines (Kazeminia et al., 2020). Given the diverse economic significance of this species, it has been widely introduced from Yili, Xinjiang, to northeastern China. However, *L. angustifolia* may be affected by cold stress in these regions. Large temperature fluctuations due to late spring frosts and their effects on *L. angustifolia* have become more frequent in the expanding cultivation areas in recent years (**Supplementary Table S1**).

While numerous studies have explored the effects of cold stress on other crops, the specific mechanisms of soluble sugar metabolism and cold tolerance in *L. angustifolia* remain poorly understood. Previous study indicates that the activation of self-protective mechanisms serves as the key process through which *L. angustifolia* acclimates to cold conditions (Kazeminia et al., 2020). This involves the activation of specific transcriptional programs, allowing the plant to establish a new balance between development and defense against stressful environments (Zhang et al., 2020). Among these, the BAHD acyltransferase family (*BAHD*) acyltransferase superfamily and the transcription factor MYC7 (*LaMYC7*) promoter in *L. angustifolia* exhibit responsiveness to low-temperature conditions (Dong et al., 2024; Zhang et al., 2024). Our recent research on the transcriptome also suggests that regulating photosynthetic genes may increase soluble sugar content in *L. angustifolia*, enhancing its cold tolerance (Li et al., 2023a). However, significant gaps still exist in our understanding of how genes related to photosynthesis and sugar metabolism function to enhance resistance in *L. angustifolia* under low temperature stress.

Little is currently understood regarding the molecular mechanisms of sugar metabolism in lavender, particularly under cold stress conditions. Therefore, the objective of this study was to investigate the key genes and their regulatory networks associated with sugar metabolism in *L. angustifolia* when subjected to cold stress. Initially, WGCNA alongside gene function annotation methods were utilized to screen key differentially expressed genes (DEGs). A co-expression network of DEGs was established using weighted correlation network analysis (WGCNA) to link physiological changes in soluble sugar metabolism to the transcriptome under cold stress. Through the functional annotation of PFAM genes, we also focused on the Glycosyl hydrolase family and the Glycosyl transferases group. Gene modules related to cold resistance were then identified and analyzed for their primary metabolic pathways and potential functions. Next, pivotal genes were identified based on their connectivity within the corresponding networks, followed by 3D structure prediction and protein docking analysis. Finally, a cold resistance network was constructed for hub genes associated with sugar metabolism in *L. angustifolia*, providing a theoretical foundation for exploring the molecular mechanisms underlying cold resistance in *L. angustifolia*.

## 2 Materials and methods

### 2.1 Material cultivation

Healthy *L. angustifolia* plants in the lavender garden at Harbin Normal University (126°32’49″-126°33’E, 45°51’50″-45°52’N) were selected for this study. When these plants are cultivated in the introduced field, temperatures range from 30°C in July (vigorous growth stage) to 0°C in late October (late growth stage). During the seasonal transition from summer to autumn, northwest winds frequently induce rapid temperature drops, with dramatic declines of up to10°C occurring within a single day. As shown in **Supplementary Table S1**, in areas focusing on lavender cultivation, temperatures have notably declined due to an unexpected late spring, while frost occurrences have risen. To clarify the mechanisms by which lavender responds to decreasing temperatures, we focused our investigation on the physiological and molecular changes that take place as the temperature decreases from 30°C to 0°C. For the above reasons, these plants were cultivated under gradient chilling treatments at 30°C (control), 20°C, 10°C, and 0°C. The plants were cultured in an artificial climate chamber (RGX250E, Tianjin, China) at 30°C for 24 hours (light/dark:12h/12h), then gradually exposed to 20°C, followed by 10°C, and finally to 0°C at a rate of 2°C/hour. They were cultured for 24 hours at each temperature, with three replicates and six plants per replicate for each treatment.

Plants were grouped to ensure that transcriptome sequencing samples were collected from the same plants. Mature leaves were weighed using an electronic analytical balance, quickly placed in liquid nitrogen, and then stored in a freezer (-80°C) for the physiological index measurement and transcriptome sequencing.

### 2.2 Measurement of soluble sugar metabolism index of *L. angustifolia*

The soluble sugar metabolism index was assessed using test kits for starch (Sta), glucose (Glu), sucrose (Suc), α-amylase activity (α-AMY), and β-amylase activity (β-AMY) from Solarbio, China, as well as maltose (Mal) from OKA, China. This assessment was conducted following the provided instructions.

### 2.3 Transcriptome analysis of *L. angustifolia*

Sample sequencing was performed using the IlluminaHiSeq2500 system by Beijing Ruibo Xingke Biotechnology. The original sequencing data can be accessed in the NCBI database under the accession number PRJNA765132. The dataset was comprised of 12 samples that were exposed to 4 treatment temperatures, with each treatment being biologically replicated three times.

To ensure the quality of data analysis, contaminated and low-quality sequences in the original RawReads were eliminated. The Agilent 2100 RNA Nano 6000 Assay Kit (Agilent Technologies, CA, USA) was used to evaluate RNA sample integrity and concentration. During quality control, reads with longer than 5 bp bases contaminated by linkers, those with over 5% N, and low-quality reads were removed. The GC content of Unigene is 41.02%, while the sequencing error rate for each base was calculated using the Phred score formula (Qphred). Notably, Q30 bases made up more than 90% of the samples. The CleanData obtained after quality control underwent bioinformatic analysis. Trinity was employed for assembling and splicing the remaining sequences to quantitatively analyze the abundance of Unigenes. Based on the genomic data of *L. angustifolia* (Li et al., 2023b), gene functional annotation analyses were conducted on the transcriptome. The genome and transcriptome originated from the same variety of lavender, ensuring that the comparative results were reliable. The Unigene sequence and predicted ORF sequence, assembled based on Trinity, were compared using Blast. Additional comparisons were made with UniProt, PFAM, and other databases, while Trinotate was used to integrate the comprehensive function annotation results. Through the functional annotation of PFAM genes, we specifically focused on the Glycosyl hydrolase family and the Glycosyl transferases group. Then, five key genes closely associated with sugar metabolism were identified in *L. angustifolia*.

### 2.4 Co-expression network analysis of *L. angustifolia*

The co-expression network of differentially expressed genes (DEGs) in *L. angustifolia* was analyzed using the WGCNA package on the Annoyun online website (https://c.solargenomics.com). The transcription data was categorized to construct a gene expression network for *L. angustifolia* under cold stress. Gene modules were identified based on clustering relationships, and co-expression modules specifically responsive to low temperatures were found by analyzing their correlation with sugar metabolism indices. Utilizing the OmicStudio tool (https://www.omicstudio.cn) and the Pearson correlation calculation method, a cluster correlation heat map with markers was generated to identify the core hub genes of *L. angustifolia* in response to low temperatures. The functional annotation analysis of DEGs with a *Q-*value ≤ 0.05 was conducted using the Gene Ontology (GO) database (http://geneontology.org/) and the Kyoto Encyclopedia of Genes and Genomes (KEGG) database (https://www.genome.jp/kegg/).

### 2.5 Protein interaction network analysis and protein structure prediction of *L. angustifolia*

The protein sequences were compared with those of *Arabidopsis thaliana* using the STRING Protein Interaction Database (http://string-db.org/). After prediction by STRING, key protein functions were validated against transcriptome Blast results, confirming consistency with STRING predictions. Key proteins were identified using Cytoscape (version 3.7.2) and the CytoHubba plug-in MCC (Maximal Clique Centrality).

ProtParam was utilized for predicting basic information about the proteins. Prabi was employed for the prediction of protein secondary structure. SMART was used to annotate the protein domains. SWISS-MODEL (https://swissmodel.expasy.org/) was applied for predicting the tertiary structure of the proteins.

We used the Ramachandran Plot from the SAVESv2.0 server to validate the three-dimensional protein model. We also employed ZDOCK3.0.2 software for protein-protein molecular docking and PDBePISA for analyzing protein-protein interaction sites. Visualization of protein-protein docking was carried out using Pymol, and Lig-plot+ software was used to analyze protein interaction forces (Laskowski and Swindells, 2011).

### 2.6 Validation of RNA-seq data by qRT-PCR

A total of fifteen DEGs, including those closely associated with photosynthesis and carbohydrate metabolism, were selected for qRT-PCR using *β-ACTIN* as a reference gene. The Primer Premier 6.0 software was utilized for primer design, and the primers used for qRT-PCR can be found in **Supplementary Table S2**.

Following the manufacturer’s instructions, total RNA was extracted from the *L. angustifolia* leaves using the MagZol™Reagent (Trizol Reagent) (Magen Biotechnology, China) and reverse transcribed using the Reverse Transciptase KIT(M-MLV) (Beijing Zomen Biotechnology, China). Subsequently, the qRT-PCR was carried out on the Mx3000P system (Agilent Stratagene) using 2×T5 Fast qPCR Mix (SYBRGreenI). The relative expression of each gene was calculated using the 2^-ΔΔCT^ method, and the experiment was conducted in triplicate.

### 2.7 Statistical analysis

The physiological data of soluble sugar metabolism were statistically processed using Excel (2016) and one-way analysis of variance (one-way ANOVA), and Student’s t-test were performed using SPSS software (IBM SPSS Statistics 25.0) to determine the differences in soluble sugar contents and their statistical significance. The statistical results of the data conform to the normal distribution. The indices of sugar metabolism were determined using a standard curve, ensuring the data obtained is accurate and reliable. The mean ± standard deviation (S.D.) was calculated from three separate experiments for statistical analysis (*P*<0.05).

The OmicStudio tool and Pearson correlation analysis (https://www.omicstudio.cn) were used to generate a cluster heat map with markers. Then, cluster heat map analyses of key genes involved in sugar metabolism and physiological indexes for soluble sugar metabolism were conducted. Correlation analyses between physiological indexes were performed using CorrPlot within the OmicStudio tools.

## 3 Results and analysis

### 3.1 Soluble sugar metabolism in responses to cold stress in *L. angustifolia*

**Figure 1:**
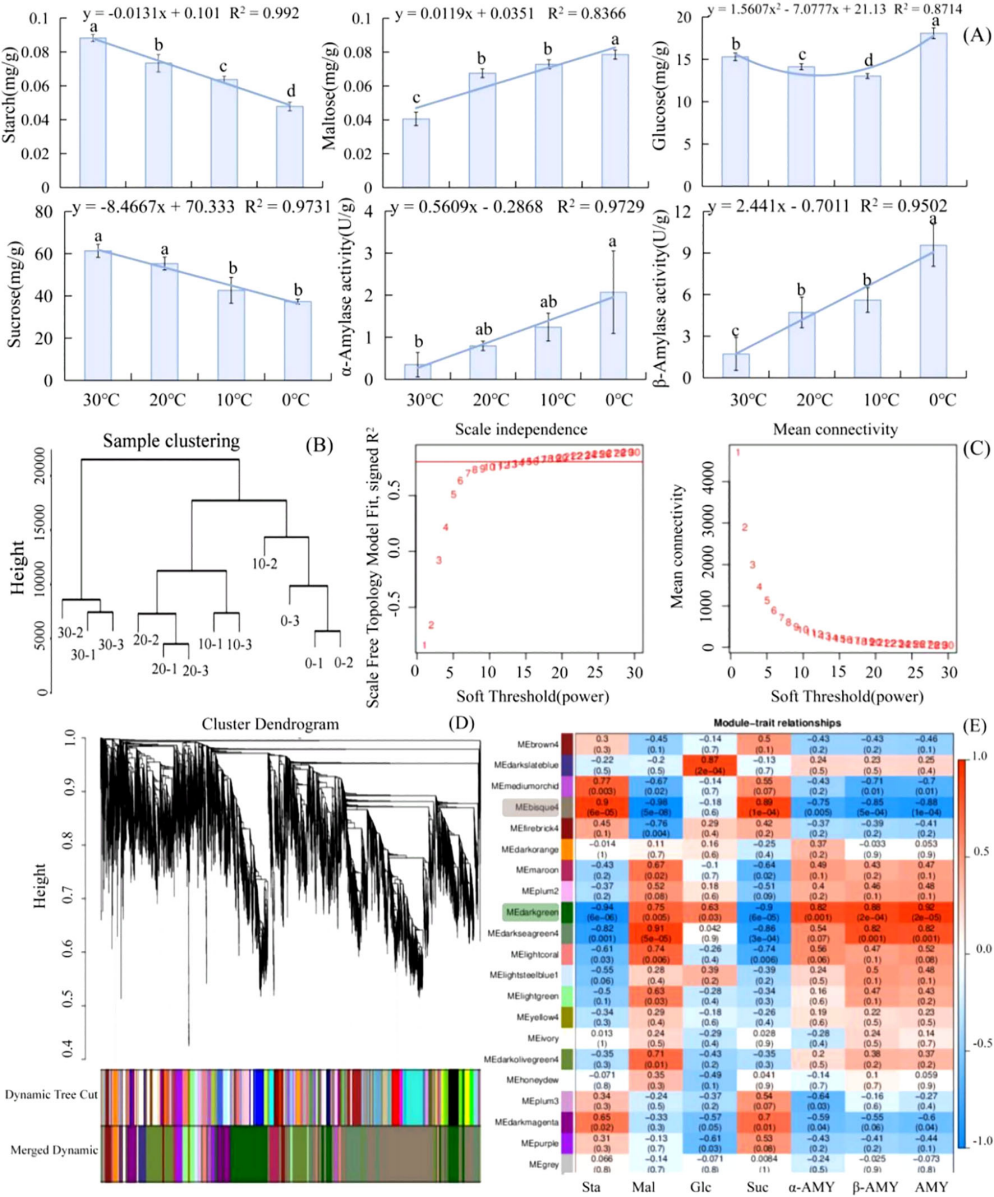
Analysis of soluble sugar metabolism and DEGs based on WGCNA in *L. angustifolia*. **(A)** The changes of soluble sugar contents and amylase activities in responses to cold stress in *L. angustifolia*. **(B)** Sample Cluster Tree; **(C)** Determination of soft threshold of gene co-expression network; **(D)** Gene clustering and module cutting in co-expression network. **(E)** Heatmap of association between co-expression network modules of genes and sugar metabolism indices. Sta, starch; Glu, glucose; Mal, maltose; Suc, sucrose; α-AMY, α-amylase activity; β-AMY, β-amylase activity; AMY, amylase. Bars represented means ± S.D. (n = 3).

As the temperature dropped from 30°C to 0°C, starch began to decompose and its contents decreased in *L. angustifolia*. There was an increase in the contents of the intermediate products glucose and maltose and a decrease in the contents of end product sucrose. These were related to an observed increase in amylase activity, leading to starch decomposition (**Figure 1A**). The regression analyses between physiological indices and temperatures revealed that the sugar metabolism of *L. angustifolia* appears to be closely linked to low-temperature stress. The changes in glucose levels under stressful temperatures follow a binomial equation trend, while the changes in other sugar metabolic indexes exhibit a linear decrease with decreased temperatures (**Figure 1A**).

### 3.2 Transcriptome analysis of *L. angustifolia*

#### 3.2.1 Co-expression network analysis of unigenes in cold stress

After performing data analysis and normalization processing, a total of 30,746 high-expression genes were identified. Sample clustering was applied to analyze 12 groups of gene samples, and the results indicated strong gene clustering under low temperatures, validating the reliability of the data (**Figure 1B**).

Further screening of weight values led us to select β=15 in the construction of the network (**Figure 1C**). The dynamic cut tree method was then applied to merge the modules with similar expressions. This resulted in a total of 21 co-expression modules (**Figure 1D**), each represented by a different color. The gene expression level was analyzed to obtain sample clustering and trait correlation through calculation. As shown in **Supplementary Figure S1**, the results demonstrated a strong correlation between the gene clustering tree and each tissue sample.

The number of genes per module was determined based on expression levels, and the genes with higher clustering degrees were assigned to specific modules. The MEbisque4 and MEdarkgreen contained the largest number of genes, with 1965 and 1280 genes, respectively. The MEgrey consisted of genes that were not assigned to any other modules.

Twenty modules and seven differential glycometabolism physiological indices (starch, glucose, maltose, sucrose, α-amylase activity, β-amylase activity, and amylase) were used in conjunction with the Pearson correlation analysis to examine any existing relationships. The findings revealed that two modules (MEbisque4 and MEdarkgreen) were correlated with the glycometabolism content in *L. angustifolia* (**Figure 1E**).

Notably, the MEbisque4 exhibited the strongest correlation with Mal (*P*<0.01) and demonstrated a significant correlation with starch, sucrose, α-amylase activity, β-amylase activity, and amylase (*P*<0.01) (**Figure 1E**). The MEdarkgreen exhibited the strongest correlation with starch, maltose, sucrose, α-amylase activity, β-amylase activity, and amylase (*P*<0.01), as well as a significant correlation with glucose (*P*<0.05) (**Figure 1E**).

#### 3.2.2 The function annotation of DEGs in *L. angustifolia*

**Figure 2:**
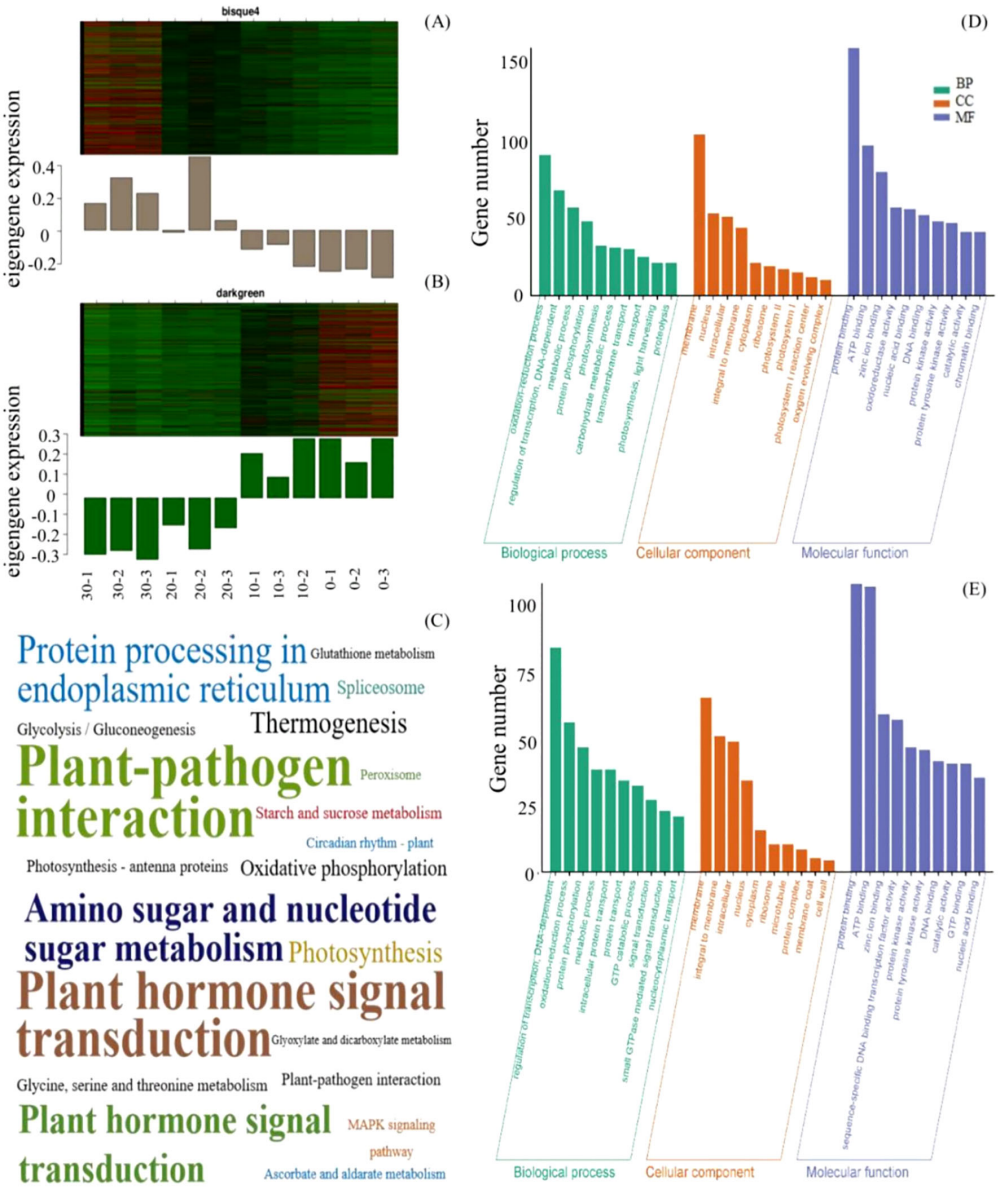
MEbisque4 and MEdarkgreen genes function analysis based on WGCNA analysis in *L. angustifolia*. **(A, B)** represented heatmap and histogram of key gene expression within the MEbisque4 and MEdarkgreen, respectively. The horizontal axis represented 3 replicates corresponding to temperatures of 30, 20, 10, and 10°C. **(C)** represented KEGG pathway within the MEbisque4 and MEdarkgreen. The KEGG enrichment factor is depicted by the size of the words in the image; a larger font size corresponds to a higher level of KEGG enrichment, thereby intuitively reflecting the significance of each KEGG pathway. **(D, E)** represented GO function enrichment within the MEbisque4 and MEdarkgreen.

The expression patterns of the MEbisque4 presented notable down-regulated expression under cold stress, whereas those within the MEdarkgreen exhibited significant up-regulated expression (**Figures 2A, B**). To determine the function of the acquired unigenes, we analyzed the enrichment of GO and KEGG in MEbisque4 and MEdarkgreen, providing annotation information about its functions. Following GO enrichment analysis (*Q*-value ≤ 0.05), we identified the most enriched GO terms. In the biological process category, the DEGs in the MEdarkgreen were significantly enriched in the regulation of transcription, the oxidation-reduction process (GO:0055114), and the metabolic process (GO:0008152) (**Figure 2E**). In the biological process category, the MEbisque4 DEGs were enriched in the metabolic process (GO:0008152) and in photosynthesis (GO:0015979) (**Figure 2D**). Notably, protein binding (GO:0005515) was the predominant molecular function category in these two modules.

In KEGG enrichment analysis, it was found that the two modules were enriched in the plant-pathogen interaction pathway and the plant hormone signal transduction pathway. Specifically, the photosynthetic pathway was enriched in the MEbisque4, while the starch, sucrose metabolism, and glycolysis/gluconeogenesis pathways were enriched in the MEdarkgreen (**Figure 2C**). The KEGG pathway results were similar to GO analysis.

**Figure 3:**
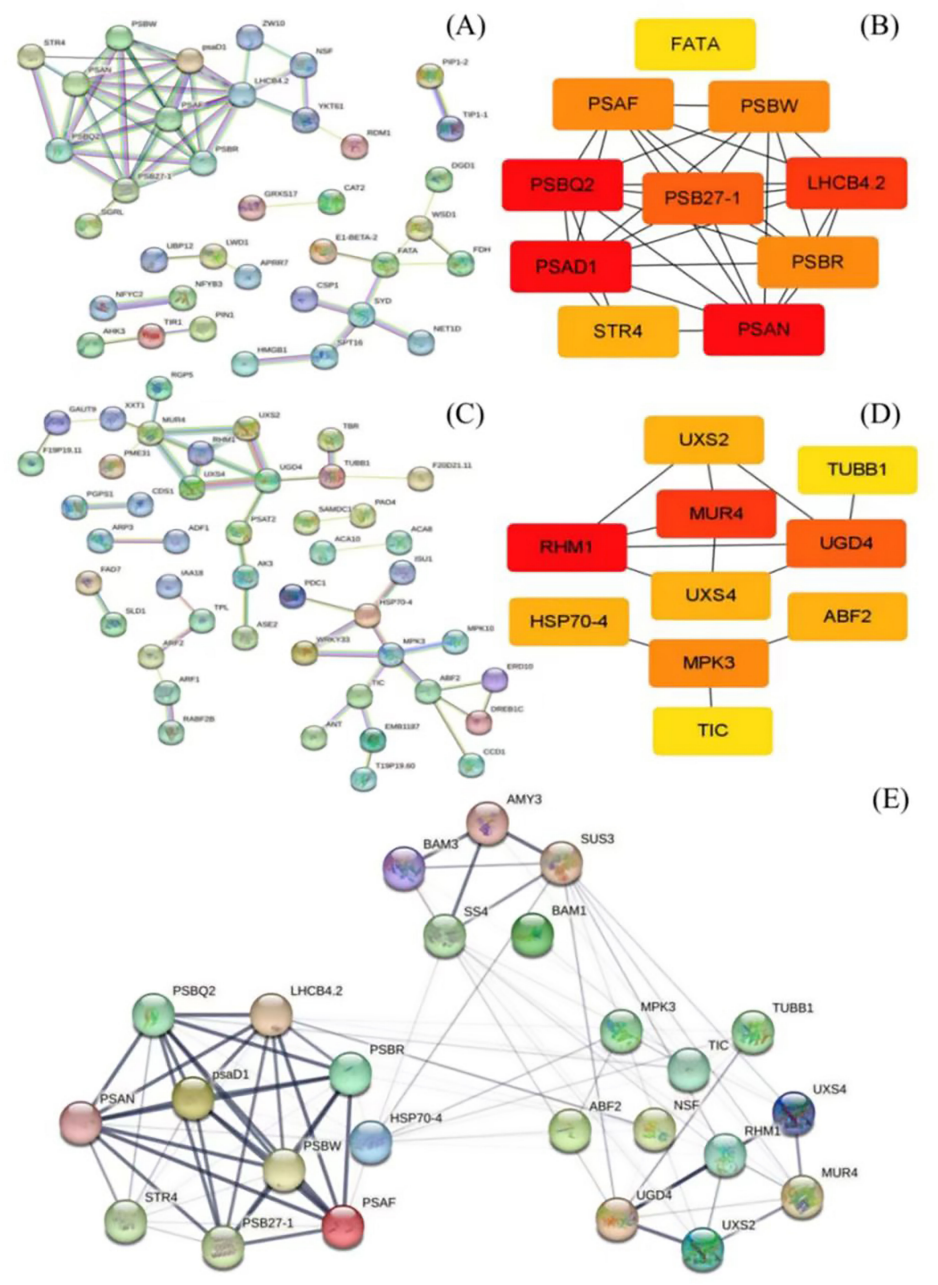
Protein network interactions and hub gene screening in *L. angustifolia* (according to *Arabidopsis thaliana* database). **(A, B)** were protein network interactions and hub gene screening in MEbisque4, while **(C, D)** were in MEdarkgreen. **(E)** Hub proteins network interactions from the MEbisque4, the MEdarkgreen and starch metabolic pathway in *L. angustifolia*. AMY3, Alpha-amylase 3; BAM3, Beta-amylase 3; SUS3, Sucrose synthase 3; SS4, Starch synthase 4; BAM1, Beta-amylase 1; LHCB4.2, Light-harvesting complex II chlorophyll a/b binding protein 4.2; MPK3, Mitogen-activated protein kinase 3; TUBB1, Tubulin beta-1 chain; PSBR, Photosystem II reaction center protein R; TIC, Translocon at the inner envelope membrane of chloroplasts; psaD1, Photosystem I reaction center subunit Il D1; UXS4, UDP-glucuronate decarboxylase 4; NSF, N-ethylmaleimide-sensitive factor; HSP70-4, Heat shock protein 70-4; RHM1, Rhamnose biosynthesis 1; PSBW, Photosystem Il reaction center W protein; STR4, Sterol 3-glucosyltransferase 4; UGD4-UDP-glucose dehydrogenase 4; PSB27-1 - Photosystem Il subunit Psb27-1; Uxs2, UDP-glucuronate decarboxylase 2; PSAF, Photosystem I subunit F.

Based on GO analysis, the KEGG pathway, and weight values in the co-expression network, pairs of network relationships between the two modules were selected to generate a co-expression network. The DEGs in the MEbisque4 and MEdarkgreen groups correspond to specific proteins, respectively. We have selected protein names that align with the model organism *Arabidopsis thaliana* from the STRING database for further investigation of protein interactions (**Figures 3A, C**). Hub genes were identified using the CytoHubba plugin, and the top 10 DEGs were selected for analysis based on their MCC score (**Figures 3B, D**). Meanwhile, we screened 5 key DEGs (*LaBAM1*/*3*, *LaAMY3*, *LaSUS3*, and *LaSS4*) that are integral to the metabolic pathways of starch and sucrose based on transcriptomic data using gene function annotation methods. We subsequently analyzed their protein interactions with hub genes in the MEbisque4, MEdarkgreen networks and starch metabolic pathway. In total, proteins were included in this analysis, which existed 92 interactions within the protein network (**Figure 3E**).

#### 3.2.3 Predictive analysis of key proteins functionality in *L. angustifolia*

The proteins LaPSAD1, LaPSAN, LaPSBQ2, LaLHCB4.2, LaPSB27-1, LaRHM1, LaMUR4, LaUGD4, LaMPK3, and LaABF2 were determined to be acidic and hydrophilic. Among them, the proteins LaPSB27–1 and LaMPK3 exhibited strong stability, while the stability of LaUGD4 was comparatively lower (**Supplementary Table S3**).

Protein domains were annotated using SMART (**Supplementary Figure S2**), revealing that LaPSAD1, LaPSAN, LaPSBQ2, LaLHCB4.2, and LABSB27–1 contain corresponding photosynthetic domains involved in photosynthesis. Furthermore, LaRHM1 and LaMUR4 were found to possess an Epimerase domain, indicating their involvement in important metabolic pathways. LaUGD4 contained three central domains of UDP-glucose (UDPG_MGDP_dh). LaMPK3 possessed a serine/threonine protein kinase (STK) domain, while LaABF2 had a BRLZ (Basic Region and Leucine Zipper, Bzip) domain. We determined that LaABF2 contained two LaPSAD1 and three low-complexity domains (LCDs), which were involved in various cellular processes, ranging from gene expression to signal transduction.

### 3.3 Analyses of key protein docking

**Figure 4:**
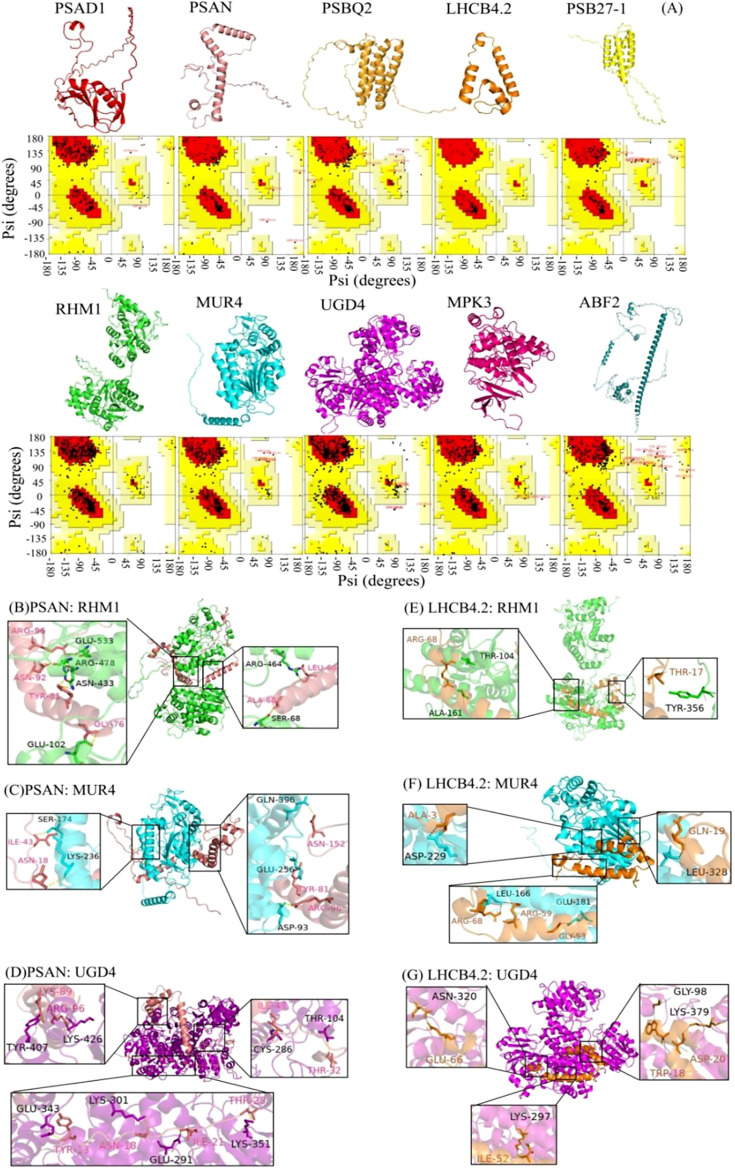
Three-dimensional structural model and protein docking diagram in *L. angustifolia*. **(A)** Three-dimensional structural model and Raman spectrum in the MEbisque4 and MEdarkgreen. Every protein corresponded to Raman spectrum below it. **(B–G)** Schematic diagram of the 3D protein docking diagram and action structure of amino acid residues.

The prediction and construction of the tertiary structures of the above proteins were performed using SWISS-MODEL. The tertiary structure model with the highest confidence (based on GMQE size) was selected. The reliability of the three-level structure model was established using SAVES v6.0 Raman spectrum analysis. This showed that more than 80% of amino acid residues fell within the most acceptable region, indicating that the ten protein models were reliable (**Figure 4A**).

The ten key proteins were categorized into photosynthetic proteins, glycometabolism proteins, and hormone proteins, with protein molecular docking predictions performed for each protein. The free energy calculations for the docking of PSAN: RHM1, PSAN: MUR4, and PSAN: UGD4, as well as LHCB4.2: RHM1, LHCB4.2: MUR4, and LHCN4.2: UGD4 showed that these interactions had the lower free energy values (**Supplementary Table S4**). This observation implies that they exhibit a higher likelihood of successful binding. The interaction sites of the protein docking complexes were analyzed using the PDBePISA online program. This program has the capability to predict the formation of hydrogen bonds and salt bridges between amino acid residues in protein-protein interactions.

The interaction sites between PSAN and RHM1, MUR4 and UGD4, as well as between LHCB4.2 and RHM1, MUR4, and UGD4 were predicted. Protein-protein docking was performed using the ZDOCK3.0.2 tool, resulting in the respective structures of the protein docking complexes. Our analysis showed that the best binding sites for these proteins were in the hydrophobic cavities of glycometabolic proteins. We also found that these glycometabolic protein molecules could provide enough space for PSAN and LHCB4.2 (**Figures 4B–G**).

Upon further examination, it was noted that a unique interaction involving distinct amino acid residues exists between PSAN and RHM1, MUR4 and UGD4 as well as LHCB4.2 and RHM1, and MUR4 and UGD4, (**Figures 4B–G**). Because of this interaction, the water presence was minimized, facilitating the formation of stable complexes. The molecular docking was bound not only by van der Waals forces and hydrogen bonds, but also by hydrophobic forces (**Supplementary Figures S3, S4**). The docking of PSAN, glycobotany proteins, and the amino acid residues Ile43, Asn18, and Arg96 were all predicted. We speculated that these sites were the main sites when glycobotany proteins docked with PSAN (**Figures 4B–D**). Similarly, we found that Arg68 was also an important locus in LHCB 4.2: RHM1 and LHCB 4.2: MUR4 (**Figures 4E–G**). The theoretical analysis of binding sites and interaction types provided structural information for our experiment.

### 3.4 Physiological and gene expression verification in *L. angustifolia*

**Figure 5:**
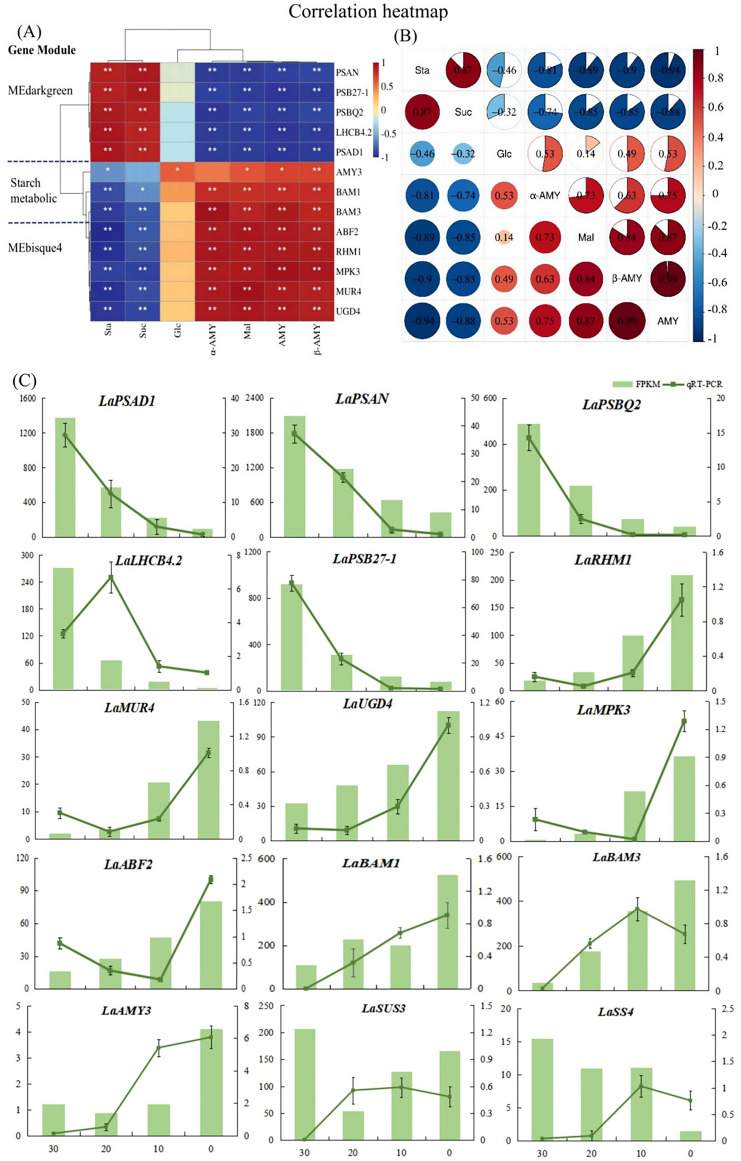
Analysis of key genes and sugar metabolism physiological indexes in *L. angustifolia*. **(A)** Heat map analysis of key genes in the MEbisque4, the MEdarkgreen and the starch metabolicbased pathway clustered with physiological indexes. Sta, starch; Glu, glucose; Mal, maltose; Suc, sucrose; α-AMY, α-amylase activity; β-AMY, β-amylase activity; AMY, amylase; **(B)** Heat map analysis between physiological indexes. *P<0.05, **P<0.01. **(C)** RNA sequencing data accuracy was verified by qRT-PCR in *L. angustifolia*. Both FPKM and qRT-PCR repeated three times, and the average value is taken for drawing.

The hub genes were classified into two distinct categories. *LaPSAD1*, *LaPSAN*, *LaPSBQ2*, *LaLHCB4.*2, and *LaPSB27–1* exhibited significant positive correlations with starch and sucrose but had negative correlation with glucose, maltose, amylase, α-amylase activity, and β-amylase activity. However, *LaAMY3*, *LaBAM1*, *LaRHM1*, *LaMUR4*, *LaUGD4*, *LaMPK3*, and *LaABF2* exhibited an opposite correlation with sugar metabolites (**Figure 5A**). Concurrently, the starch metabolism pathway and the MEbisque4 genes displayed a similar relationship with glucose metabolic processes, suggesting that the MEbisque4 genes are closely associated with glucose metabolism. These findings are consistent with the WGCNA module and effectively demonstrate the reliability of the gene clustering analysis.

Through the correlations among physiological indexes, we observed that starch content was strongly correlated with sucrose and maltose contents, as well as with amylase, α-amylase activity, and β-amylase activity under low-temperature stress (*P*<0.01, *P*<0.05) (**Figure 5B**). Specifically, there was a positive correlation between starch and sucrose, and a negative correlation between starch and maltose, as well as between starch and amylase activities (*P*<0.01, *P*<0.05). Sucrose showed a significant correlation with maltose and amylase activities, and maltose was positively correlated with amylase activities, suggesting that the decomposition of starch and the synthesis of sucrose were closely related to AMY activity under low-temperature stress in *L. angustifolia*. In contrast, glucose, as an intermediate, had little effect under low-temperature stress.

To validate the accuracy of the RNA-seq results, fifteen DEGs were selected for qRT-PCR verification—five genes from the starch metabolic pathway and five each from MEbisque4 and MEdarkgreen. The expression levels of *LaAMY3* and *LaBAM1*/*3* exhibited an increasing trend, which facilitated the decomposition and formation of starch. The overall expression of *LaSUS3* and *LaSS4* were increased. It is speculated that *LaSUS3* plays a role in the reversible conversion process of sucrose to glucose and fructose in *L.angustifolia*, thus promoting an increase in glucose levels in response to cold stress. The quantitative detection findings for these genes were generally consistent with the sequencing data, indicating a high level of reliability in our sequencing results (**Figure 5C**).

## 4 Discussion

Plants possess the capacity to accumulate compatible solutes at low temperatures as a result of adaptive evolutionary processes (Zuther et al., 2012). When plants are exposed to cold stress, the total sugar contents can increase by changes of the enzyme activities and acceleration of the starch-sucrose pathway (Li et al., 2023a). These processes cause starch to decompose into glucose and maltose, resulting in sugar accumulation (Zhang et al., 2017a). Meanwhile, the sugars serve as a protective agent for plant cells at low temperature by reducing cell osmotic potential and scavenging active oxygens to increase the cold resistance (Bai et al., 2023). Our study found that as the temperature dropped from 30 °C to 0 °C, the amylase activities significantly increased, while starch contents reduced (**Figure 1A**). This drop in temperature corresponded with elevated levels of maltose and glucose levels, which were closely associated with heightened amylase activity. This accelerated starch breakdown, providing plants with the energy to adapt to cold conditions and maintain cellular homeostasis and physiological processes (Zhang et al., 2017a).

Plant adaptation to low-temperature stress is governed by intricate physiological and molecular regulatory mechanisms (Liu et al., 2021). Using WGCNA analysis, we divided the transcriptome data into distinct gene co-expression modules, with genes in the same module exhibiting similar expression patterns and carrying out comparable biological functions. We obtained specific MEbisque4 and MEdarkgreen in *L. angustifolia* at low temperatures, and these modules showed close associations with sugar metabolism and demonstrated high levels of gene clustering (**Figure 1E**). Five key genes involved in the starch metabolism pathway were also identified, along ten key genes present in MEbisque4 and MEdarkgreen modules. Our findings demonstrated a significant correlation between these key genes and glycometabolism content in *L. angustifolia* (**Figure 5A**). This established an important foundation for investigating the key genes associated with soluble sugar metabolism and constructing the regulatory network of soluble sugar metabolism. Various molecular pathways could aid plants in acclimating to cold environments by regulating the expression of a series of cold-response genes (Bian et al., 2022). We specifically identified *LaPSB27*, *LaPSBQ*, *LaPSAD1*, *LaPSAN*, *LaLHCB4.2*, *LaRHM1*, *LaMUR4*, and *LaUGD4* as potential core genes involved in sugar metabolism for adapting to cold stress in *L. angustifolia*. These genes are involved in the transcription regulation, metabolic processes, photosystem I and II, protein binding, DNA-dependent processes, oxidation-reduction processes, and membrane functions.

The down-regulation of *PSB27* has been observed in *Arabidopsis* under low-temperature stress, resulting in reduced efficiency of PSII while simultaneously providing protection to this complex (Hou et al., 2015). In this study, the expression of *LaPSB27* decreased under cold stress, suggesting its protective role in *L. angustifolia*. Like PSB27, PSBQ has a four-helix bundle tertiary structure and shares a similar binding site, further signifying their close relationship (Gisriel and Brudvig, 2022). The removal of *PSBQ* alone in *Arabidopsis* reduced light-harvesting complexes and altered state transitions (Allahverdiyeva et al., 2013). This suggests that *L. angustifolia* could modulate the energy flow within the PSII system through regulating *LaPSBQ* expression, thereby contributing to photoprotection (Ojosnegros et al., 2022). Under cold stress, *PSAD* could down-regulate and block the electron transfer chain, causing the inhibition of PSI (Chitnis et al., 1997). This led to a reduction in photosynthetic activity and triggered a series of protective mechanisms to maintain biological activities (Dong et al., 2022). Similarly, our study observed a down-regulation of *LaPSAD1* associated with PSI, which suggests its potential protective role in *L. angustifolia* under cold stress. Meanwhile, the expressions of *LaPSB27*, *LaPSBQ*, and *LaPSAD1* were negatively correlated with the activities of glucose, maltose, amylase, α-amylase activity, and β-amylase activity in our study (**Figure 5A**). The down-regulation in gene expression due to lower temperatures enhanced starch synthesis and its conversion into soluble sugars, boosting *L. angustifolia*’s resistance to cold.

LHCB is a crucial component of the photosynthetic antenna system, playing a pivotal role in both photosynthesis and stress response regulation (Liu et al., 2021). Under low-temperature stress (4°C), down-regulation of *PbrLhcbs* resulted in a reduced chlorophyll content and light energy utilization, allowing plants to partially adapt to cold environments (Li et al., 2023c). We found that the expression of *LaLHCB4.2* decreased under cold stress, suggesting its protective role in *L. angustifolia*’s adaptation to low temperatures. *LaPSAN* were also found to be down-regulated under cold stress, consistent with the results in pepper. PSAN is responsible for mediating LHCII energy transfer to the PSI core, and it is speculated that the LaPSAN protein in PSI plays a significant role in the response of *L. angustifolia* to cold stress (Vanselow et al., 2009; Liu et al., 2021).

A recent study has revealed that *RHMs* can directly regulate the expression of key enzyme genes in the glycolytic pathway, impacting glucose decomposition and energy production (Crowe et al., 2024). Our findings indicate that the up-regulating *LaRHM1* may enhance energy production at low temperatures in *L. angustifolia*. UGD is an essential enzyme involved in the biosynthesis of UDP-GlcA (Siddique et al., 2014), an important precursor for plant cell wall polysaccharides. In this study, we proposed that the upregulation of *LaUGD4* contributed to the protection of *L. angustifolia*’s cell wall under cold stress. MUR4 could be involved in converting UDP-D-xylose to L-Ara, thus regulating the content of Ara in the cell wall (Burget et al., 2003). In this study, the expression of the *LaMUR4* was significantly up-regulated as temperatures decreased, promoting the formation of protective cell wall substances at low temperatures (**Figure 5C**).

As previously demonstrated, *MUR4* is involved in ABA signal transduction in *Arabidopsis* (Yan et al., 2020). ABF2 contributes to stress tolerance of mature plants in the ABA signaling pathway (Yoshida et al., 2010). Our results suggest that LaMUR4 and LaABF2 function synergistically in *L. angustifolia* response to low temperatures. We also identified the involvement of MAPK pathways, which are indirectly associated with sugar metabolism during adaptation to cold. The MAPK cascade mediates plant signal transduction under environmental stress. Overexpression of *SIMPK3* significantly enhances the soluble sugar content and cold tolerance of transgenic tomatoes (Yu et al., 2015). Additionally, we observed an increase of *LaMPK3* expression after exposure to low temperatures, providing further protection for *L. angustifolia*.

Protein-protein interactions are crucial for energy, substance metabolism, and gene expression regulation. Our interaction analysis revealed a strong genetic correlation between the MEbisque4 and MEdarkgreen modules from WGCNA (**Figure 3E**), supporting finding from previous research (Chen et al., 2021). We then utilized computational techniques to generate atomic structure models of protein-protein complexes and efficiently deduced molecular functions in cells through the docking models (Sanyal et al., 2021).

In the protein docking analysis, we identified specific interaction sites involving multiple amino acid residues between LaPSAN and LaRHM1, LaMUR4 and LaUGD4 (**Figures 4B–D**), as well as between LaLHCB4.2 and LaRHM1, LaMUR4, and LaUGD4 (**Figures 4E–G**). These interactions contributed to protein stability and may also impacted biological function and activity. Among the six protein-docking results, the residues Ile43, Asn18, Arg96, Arg68, and Tyr81 were frequently found at the binding site. The ion pairing between these residues may decrease the rate constant of intermolecular charge transfer, thereby reducing energy conversion in plants (Qian et al., 2019). This reduction is beneficial for mitigating light damage at low temperatures (Yuan et al., 2021). The proteins interacting with LaPSAN and LaLHCB4.2 play a pivotal role in regulating glucose metabolism and cellular protection, highlighting the significance of photosynthetic regulation in enabling plants to withstand low temperatures. These docking results also facilitate the prediction of protein complex structures and the development of detailed metabolic pathways.

**Figure 6:**
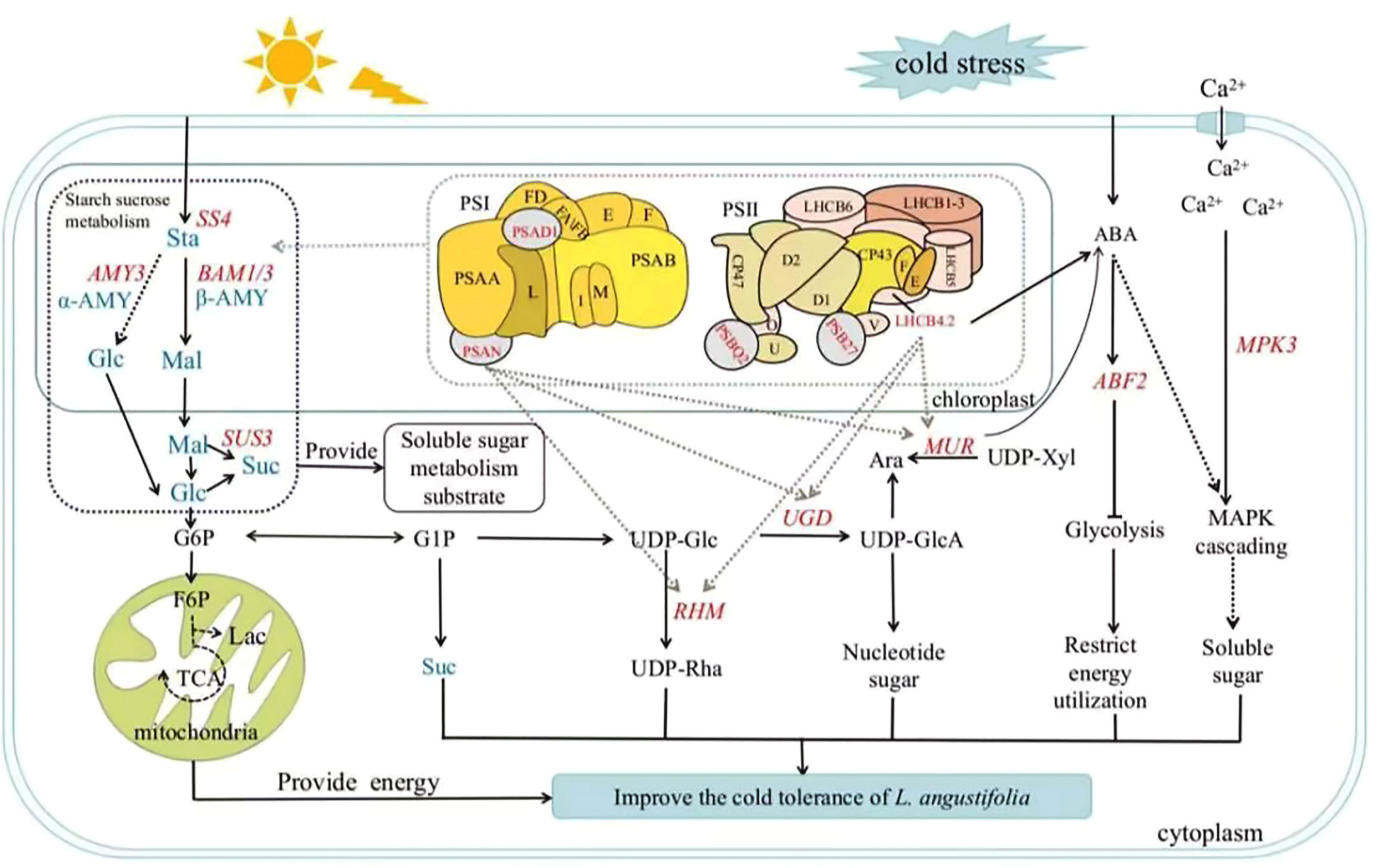
Soluble sugar metabolic pathway in *L. angustifolia*’s adaptation to low temperatures. Sta, starch; Glu, glucose; Mal, maltose; Suc, sucrose; α-AMY, α-amylase activity; β-AMY, β-amylase activity; AMY, amylase. The blue fonts represented the starch and sucrose metabolic pathway. The red fonts represented key genes that regulated sugar metabolism. The black solid and dashed lines represented direct and indirect actions, respectively. The dashed gray lines depicted molecular docking interactions between proteins. “T” solid line represented inhibitory action.

Photosynthesis is crucial for plants to produce soluble sugar, but cold stress can hinder this process and impact sugar metabolism (Ding et al., 2023). Our findings align with previous research that highlight the role of transcriptional regulation of glycometabolism genes in plants adaptation to cold stress (Liu et al., 2021). As shown in **Figure 6**, when *L. angustifolia* is exposed to cold stress, starch decomposes into glucose and maltose through amylase LaAMY and LaBAM1/3. Glucose then circulates through G6P and TCA pathways to yield G1P and sucrose (Yuan et al., 2024). Simultaneously, G1P generates UDP-Rha and UDP-GlcA via the intermediate UDP-Glc, facilitated by RHM and UDG enzymes that influence cell wall composition (Zhao et al., 2020; Mason et al., 2023).Furthermore, Ara synthesis occurs from UDP-Xyl under MUR catalysis (Dugard et al., 2016; Saez-Aguayo et al., 2017).

Plants optimize sugar metabolism for energy production, cellular homeostasis, and enhanced cold tolerance by modulating gene expression. Our analysis of protein-protein interactions revealed that hub genes form complex regulatory networks with other proteins, indicating a coordinated process involving multiple genes and proteins in sugar metabolism under cold stress. Our experiment also verified the correlation between gene expression and physiological indicators, providing deeper insights into *L. angustifolia*’s adaptability to cold stress.

## 5 Conclusions

In summary, this study shows that *L. angustifolia* adapts to cold stress by regulating its sugar metabolism and suppressing photosynthesis. As temperatures decreased from 30°C to 0°C, the degradation of starch into soluble sugars (maltose and glucose) significantly increased (*P*<0.05). This is attributed to elevated activities of α-amylase and β-amylase, closely associated with the upregulation of *LaAMY* and *LaBAM1*/*3* genes, which aid in osmotic regulation and energy supply. Transcriptomic analyses revealed an up-regulation of sugar metabolism genes (*LaRHM1*, *LaMUR4*, and *LaUGD4*) alongside a down-regulation of photosynthesis-related genes (*LaPSAD1*, *LaPSAN*, *LaPSBQ2*, *LaLHCB4.2*, *LaPSB27-1*), indicating a metabolic shift toward stress mitigation. Molecular docking predictions suggest interactions between sugar metabolism proteins (RHM1, MUR4, UGD4) and photosynthesis components (PSAN, LHCB4.2), implying a coordinated mechanism for balancing energy allocation while minimizing light-induced damage under cold conditions. These findings highlight the importance of starch-to-sugar conversion and transcriptional regulation in enhancing cold tolerance in *L. angustifolia*. This mechanistic insight is vital for breeding cold-resistant lavender varieties and optimizing cultivation strategies in low-temperature regions such as Northeast China, thus supporting agricultural productivity and economic development.

## Data Availability

The original sequencing data can be accessed in the NCBI database under the accession number PRJNA765132.
