# Supplementary material for: Investigation of the physiological and molecular regulatory mechanism of soluble sugar metabolism in Lavandula angustifolia Mill. under cold stress
Source: Front Plant Sci. 2025 Jul 8;16:1537516. doi: 10.3389/fpls.2025.1537516 (PMC12279718; doi:10.3389/fpls.2025.1537516)
Supplement: Supplementary file 1 [file DataSheet1.docx]

Supplementary Material

# Supplementary **Table**

**Table S1** Spring cold index in *L. angustifolia* growing regions in China

|  | Year | Cold snap index  (K) | Temperatures  (℃) | Temporal duration (Date) |
| --- | --- | --- | --- | --- |
| Harbin, Heilongjiang Province  126°32'-126°33'E, 45°51'-45°52'N | 2022 | 2.90 | -7.39‒3.12 | 3.06‒3.16 |
|  | 2020 | 3.55 | 0.03‒18.64 | 4.20‒5.02 |
|  | 2019 | 2.41 | -6.53‒9.09 | 3.19‒4.02 |
|  | 2017 | 2.37 | 9.71‒25.37 | 4.03‒4.08 |
| YIli, Xinjiang Region  80°11′-81°24′E, 43°39′-44°50′N | 2023 | 2.85 | 8.00‒18.00 | 4.22‒5.01 |
|  | 2022 | 3.24 | 0.90‒8.30 | 3.20‒3.25 |
|  | 2021 | 3.06 | 4.78‒15.38 | 4.21‒4.25 |
|  | 2018 | 3.53 | 8.03‒23.86 | 4.29‒5.03 |

*Note*: K=δT1÷4-δT2÷2+L÷10

K: meteorological indicator of inversion; δT1: degree of warmth in the first period; δT2: degree of coldness in the second period; L: duration of the inversion process.

**Table S2** Primers used for qRT-PCR in *L. angustifolia* leaves.

| NO. | Gene Name | Sequence of primer(5′-3′) |
| --- | --- | --- |
| 1 | *PSAD1* | F: CGGCTCCGGTCCAAATACAA |
|  |  | R: TCATAGACTTGCTTGCCGGT |
| 2 | *PSAN* | F: TCATAAGGGCTCAGGCCAGT |
|  |  | R: ATTCAGCTCCTTGTTCGCCT |
| 3 | *PSBQ2* | F: CCTTCGGTCGAAAGCAGAGT |
|  |  | R: TCGTTGAGCGCAGTTACAGT |
| 4 | *LHCB4.2* | F: GGAATCACATGGCAAGACGC |
|  |  | R: GCGTTCCTCTGGAACTCGAT |
| 5 | *PSB27-1* | F: AAGGTCCGAACCACCATCAC |
|  |  | R: GTCGGCCCGAAGCTAATGTA |
| 6 | *RHM1* | F: CACCCTGAAGGTTCTGGCAT |
|  |  | R: GACTGTCATGCTGTTGGGGA |
| 7 | *MUR4* | F: TGGAGGCTTGTAAGAAGGCG |
|  |  | R: CAGACATCACGAAAGGGGCT |
| 8 | *UGD4* | F: AAAGACACCGGTGACACGAG |
|  |  | R: CGCACTGCACTTTCTTCACC |
| 9 | *MPK3* | F: TATACGGCAGCTTCCACGAC |
|  |  | R: TGTCAGCATTTGGCTCTCCA |
| 10 | *ABF2* | F: TATACGGCAGCTTCCACGAC |
|  |  | R: TGTCAGCATTTGGCTCTCCA |
| 11 | *AMY3* | F: GCCAATCGAAAAGTTGCAACTCG |
|  |  | R: GACGCTTCAAGTCGAAAGTTCCAC |
| 12 | *BAM1* | F: ATTATGATGGATGTGTGGTGGG |
|  |  | R: TTGGGCAGAGGAATCGTGC |
| 13 | *BAM3* | F: TTGCTCACATAAAACCGTCAT |
|  |  | R: CCGTTGGTGAAAGTTGGGCTTCGTA |
| 14 | *SUS3* | F: CAACTTGAGTCTGCTCTGGGG |
|  |  | R: CCAACCTCTCTCAAAACCCATT |
| 15 | *SS4* | F: CTATGGAGCGGTTCCTGTGGT |
|  |  | R: CTCTCCATTCATCAGGCTTCTCTC |
| 16 | *β-ACTIN* | F: TGTGGATTGCCAAGGCAG |
|  |  | R: AATGAGCAGGCAGCAACA |

**Table S3** Basic information of key proteins in the MEbisque4 and MEdarkgreen based on WGCNA in *L. angustifolia*.

| Name | Module | Number of aminoacids(nos.) | Molecular weight(kDa) | Isoelectric point(pI) | Grand average of hydropathicity(GRAVY) | Aliphatic index | Instability index | α-helix | β-turn | Random-coil | Prediction of subcellular localisation |
| --- | --- | --- | --- | --- | --- | --- | --- | --- | --- | --- | --- |
| psaD1 | bisque | 645 | 54.05 | 5.06 | 1.07 | 21.09 | 41.58 | 28.04% | 19.16% | 47.20% | Chloroplast |
| PSAN | bisque | 471 | 39.02 | 5.16 | 0.92 | 23.99 | 45.07 | 36.54% | 21.15% | 35.90% | Thylakoid |
| PSBQ2 | bisque | 681 | 56.12 | 5.10 | 0.93 | 25.70 | 48.66 | 54.42% | 9.29% | 31.86% | Thylakoid |
| LHCB4.2 | bisque | 222 | 18.06 | 5.30 | 0.83 | 21.62 | 46.43 | 39.73% | 13.70% | 42.47% | Chloroplast |
| PSB27-1 | bisque | 531 | 44.55 | 5.10 | 1.07 | 21.47 | 65.91 | 53.98% | 1.14% | 38.07% | chloroplast |
| RHM1 | darkgreen | 2031 | 167.26 | 4.93 | 0.78 | 28.75 | 43.98 | 37.28% | 6.80% | 39.79% | Endoplasmic reticulum |
| MUR4 | darkgreen | 1254 | 103.12 | 5.04 | 0.75 | 28.39 | 41.29 | 35.49% | 7.19% | 41.25% | Golgo apparatus |
| UGD4 | darkgreen | 1443 | 118.21 | 5.00 | 0.73 | 26.54 | 40.29 | 43.54% | 9.17% | 30.00% | chloroplast |
| MPK3 | darkgreen | 1029 | 85.41 | 5.04 | 0.85 | 26.43 | 56.00 | 37.28% | 6.56% | 40.62% | cytoskeleton |
| ABF2 | darkgreen | 1245 | 98.56 | 5.05 | 0.70 | 27.31 | 43.13 | 29.95% | 1.93% | 59.42% | Nuclear |

**Table. S4** Basic information of key proteins in the MEbisque4 and MEdarkgreen molecular docking model prediction between protein and protein in *L. angustifolia*.

| **Proteins name** | **SurfaceÅ2-1** | **SurfaceÅ2-2** | **Interface area, Å2** | **ΔiGkcal/mol** | **N_HB_** | **N_SB_** |
| --- | --- | --- | --- | --- | --- | --- |
| PSAD1:RHM1 | 18878 | 34856 | 3134.5 | -16.2 | 21 | 8 |
| PSAD1:MUR4 | 18878 | 23485 | 3331.3 | -23.9 | 20 | 2 |
| PSAD1:UGD4 | 18878 | 21976 | 190 | -0.8 | 1 | 2 |
| PSAN:RHM1 | 15920 | 34832 | 2853 | -30.2 | 13 | 5 |
| PSAN:MUR4 | 15920 | 23470 | 2298.1 | -26.2 | 7 | 5 |
| PSAN:UGD4 | 15920 | 21948 | 2361.7 | -27.1 | 11 | 4 |
| PSBQ2:RHM1 | 20702 | 34815 | 2412.2 | -24.2 | 9 | 5 |
| PSBQ2:MUR4 | 20702 | 23451 | 2509.7 | -15.2 | 8 | 3 |
| PSBQ2:UGD4 | 20702 | 21952 | 1897 | -12 | 9 | 4 |
| LHCB4.2:RHM1 | 6802 | 34821 | 1397 | -20.6 | 5 | 0 |
| LHCB4.2:MUR4 | 6802 | 23484 | 1527.7 | -24.8 | 9 | 1 |
| LHCB4.2:UGD4 | 6802 | 21976 | 978.8 | -11.8 | 4 | 0 |
| PSB27-1:RHM1 | 15391 | 34842 | 2164.8 | -17.1 | 11 | 12 |
| PSB27-1:MUR4 | 15391 | 23479 | 1281.6 | -11.7 | 3 | 0 |
| PSB27-1:UGD4 | 15391 | 21937 | 1710.3 | -12.1 | 5 | 2 |
| MPK3:RHM1 | 18212 | 34843 | 2780.6 | -20.2 | 22 | 9 |
| MPK3:MUR4 | 18212 | 23472 | 1772.6 | -15.7 | 13 | 3 |
| MPK3:UGD4 | 18212 | 21955 | 1481.8 | -6.3 | 10 | 2 |
| ABF2:RHM1 | 45425 | 34833 | 2183.0 | -12.6 | 11 | 3 |
| ABF2:MUR4 | 45425 | 23469 | 2040 | -14.4 | 10 | 0 |
| ABF2:UGD4 | 45425 | 21951 | 1694.7 | -7.8 | 12 | 0 |

*Note*: Surface Å2-1 and surface Å2-2 represented the total solvent accessible surface area in square Ångstroms concerning the docking proteins. Interface area in Å2 calculated as difference in total accessible surface areas of isolated and interfacing structures divided by surface Å2-2. ΔiG represented the solvation free energy gain upon formation of the interface. N_HB_ and N_SB_ represented the number of potential hydrogen bonds across the interface and salt bridges across the interface, respectively.

# **Supplementary Figure**


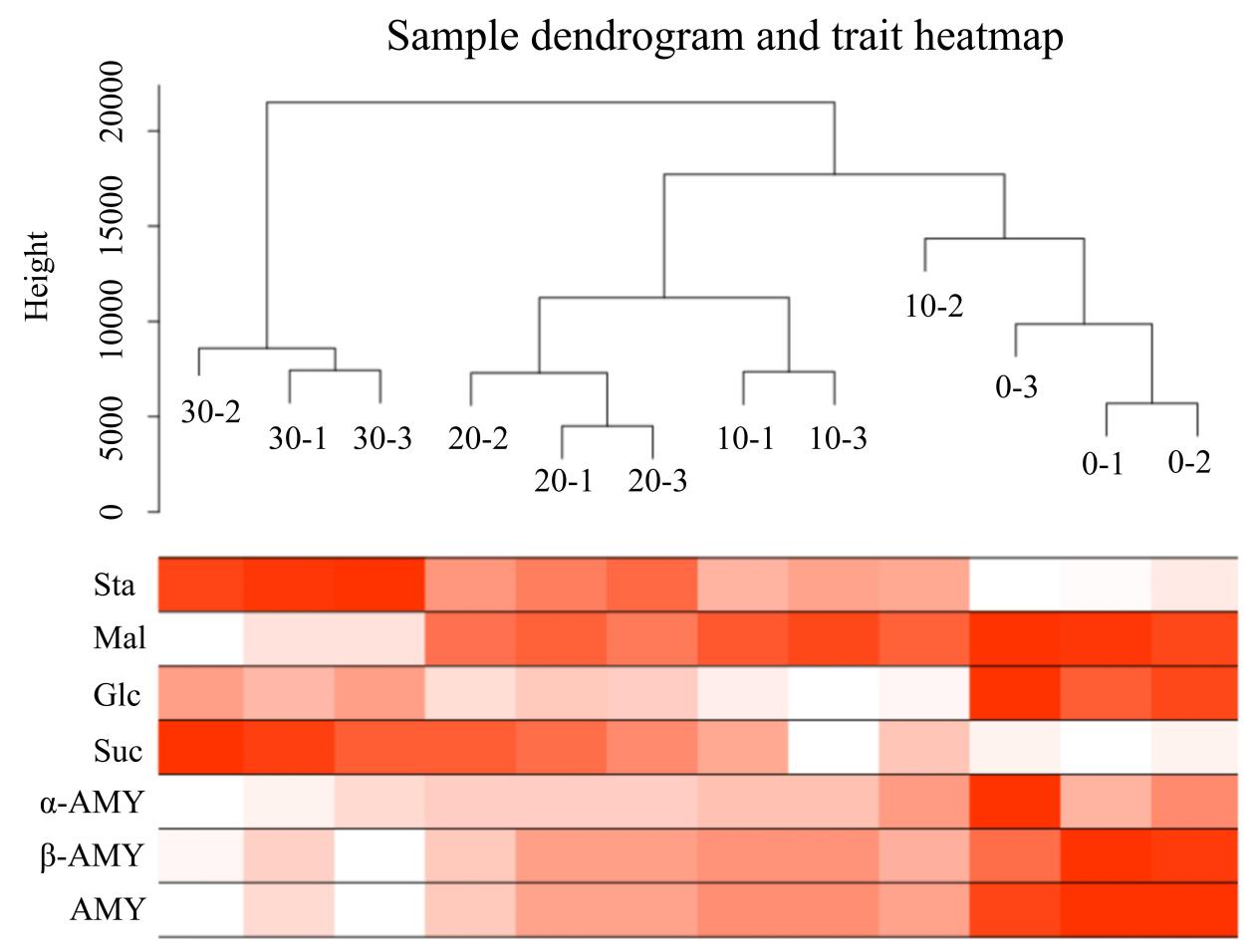


**Figure. S1** The heatmap between sample cluster tree and sugar metabolism indexes in *L. angustifolia*.

*Note:* Sta: starch, Glu: glucose, Mal: maltose, Suc: sucrose, α-AMY: α-amylase activity, β-AMY: β-amylase activity, AMY: amylase. 30-1, 30-2, 30-3, 20-1, 20-2, 20-3, 10-1 10-2, 10-3, 0-1, 0-2, and 0-3 represented 3 replicates of 30, 20, 10, and 0 ℃, respectively.

**
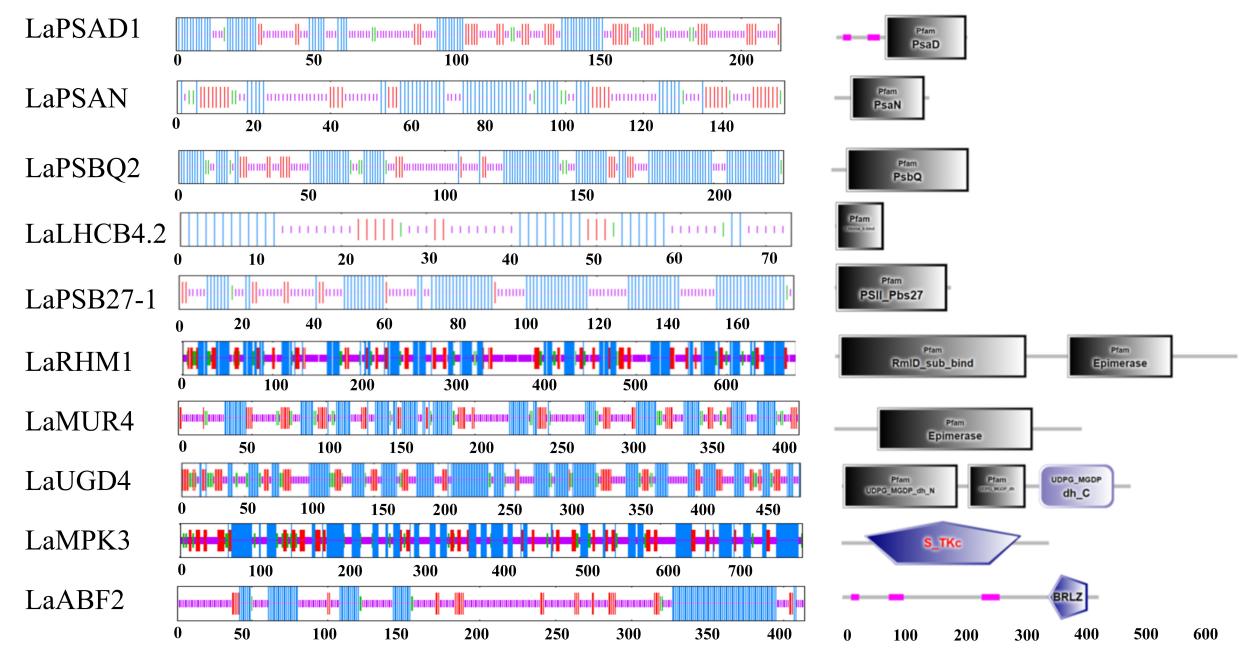
**

**Figure. S2** Two-dimensional structural model and protein structural domain in the MEbisque4 and MEdarkgreen based on WGCNA in *L. angustifolia*.

*Note*: Blue: α-helix, Green: β-turn, Purple: Random coil; Red: Extended strand.


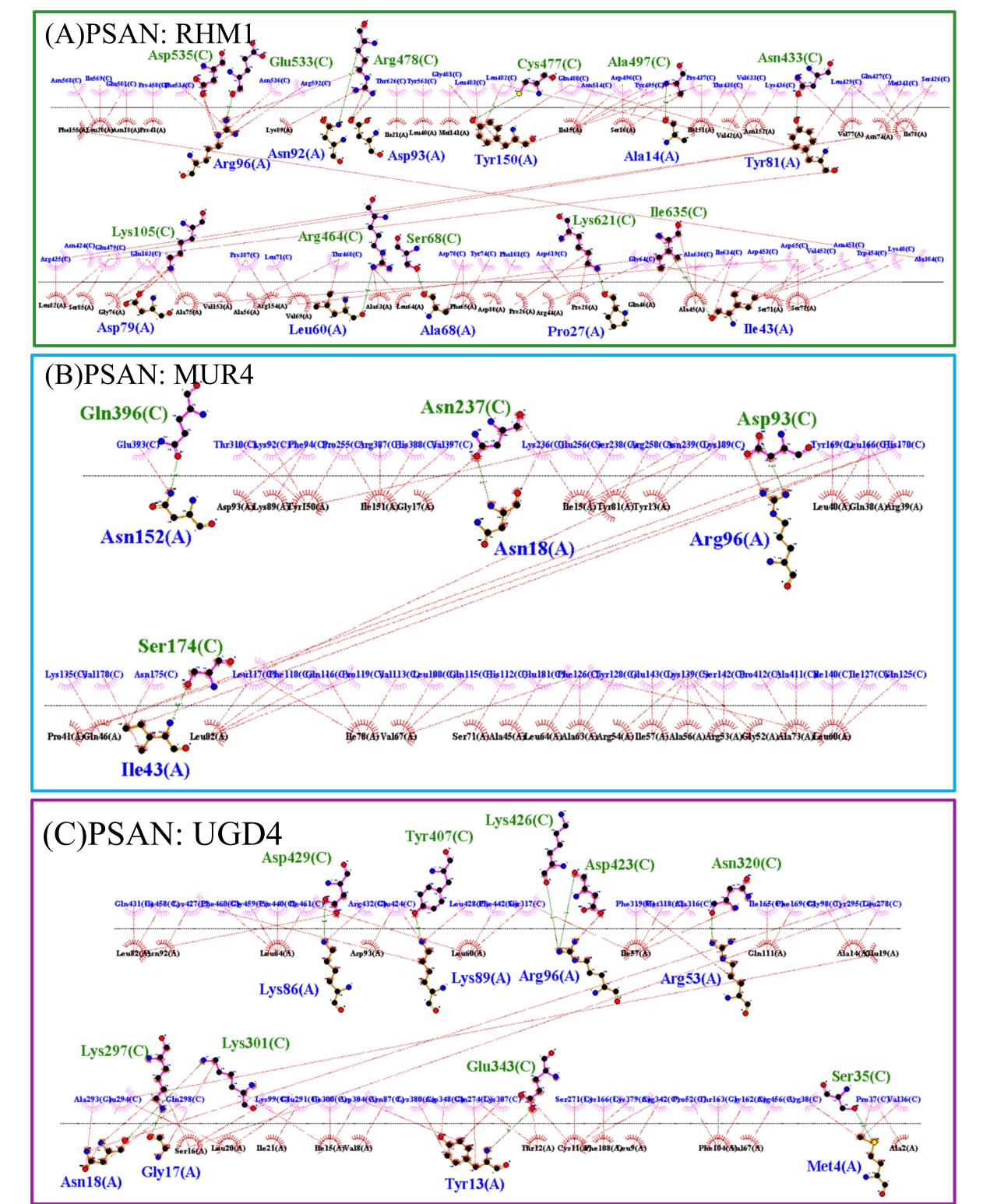


**Figure. S3** The 2D diagram of the interaction between PSAN and protein amino acid residues in *L. angustifolia*.


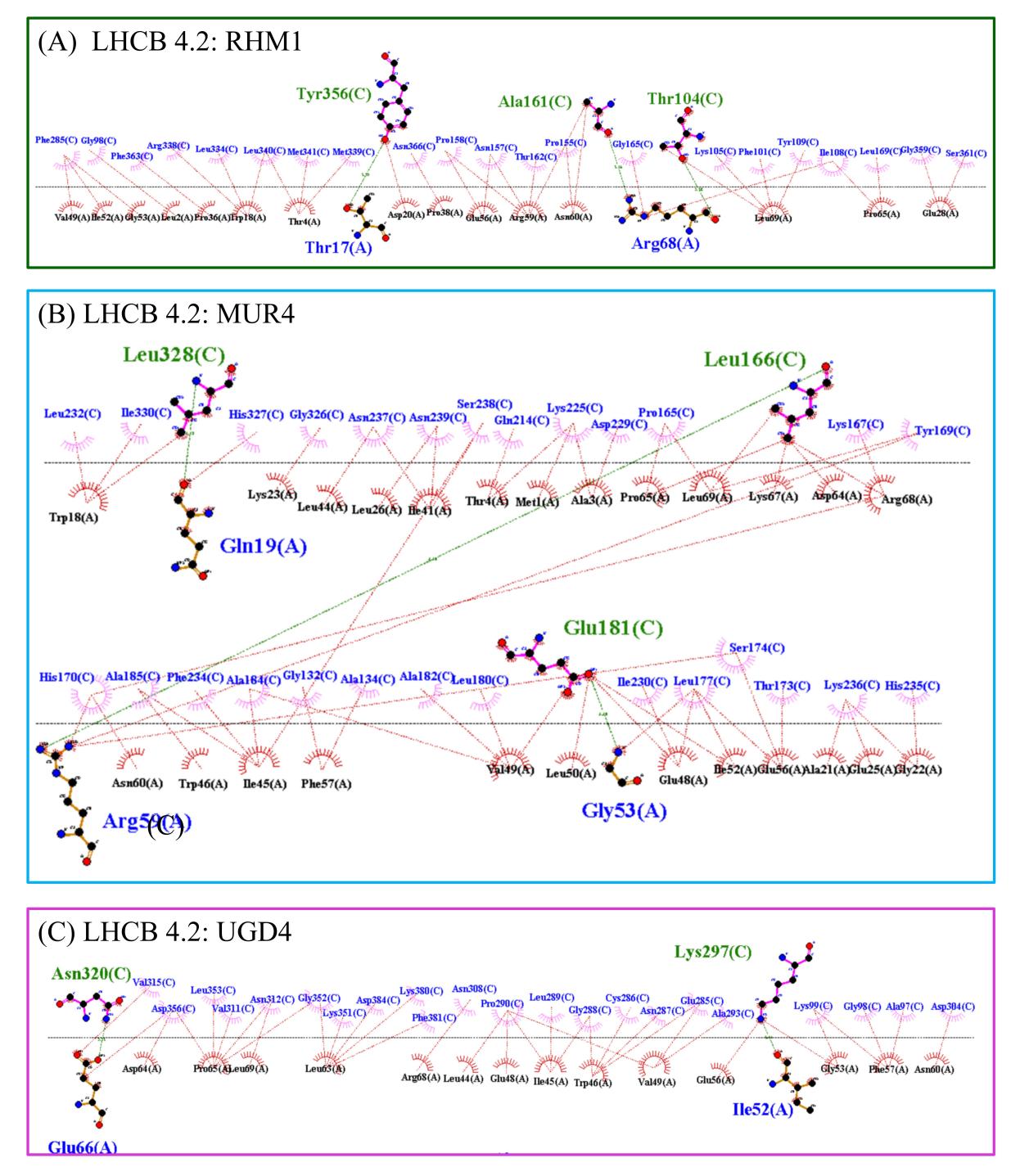


**Figure. S4** The 2D diagram of the interaction between LHCB 4.2 and protein amino acid residues in *L. angustifolia*.
